# rFIP-nha activates macrophages towards a pro-inflammatory phenotype via AIM2 inflammasome modulation

**DOI:** 10.3389/fcell.2025.1533742

**Published:** 2025-04-28

**Authors:** Yusi Liu, Zhen Li, Harry Wichers, Shanna Bastiaan-Net, Tamara Hoppenbrouwers

**Affiliations:** ^1^ Wageningen Food and Biobased Research, Wageningen University and Research, Wageningen, Netherlands; ^2^ Laboratory of Food Chemistry, Wageningen University, Wageningen, Netherlands; ^3^ Laboratory of Biomanufacturing and Food Engineering, Institute of Food Science and Technology, Chinese Academy of Agriculture Sciences, Beijing, China; ^4^ Laboratory of Food Quality and Design, Wageningen University, Wageningen, Netherlands

**Keywords:** immunomodulatory protein, macrophage, pro-inflammatory, IL-1β, AIM2 inflammasome, glycosylation

## Abstract

Fungal immunomodulatory proteins (FIPs) are small proteins from fungi with considerable immunomodulatory activity. FIP-nha (*Nectria haematococca*) contains two glycosylation sites at positions N5 and N39, and displays a high thermostability and notable anti-tumour activity. However, FIP-nha’s immunomodulatory activity on macrophages and the associated mechanism remain unclear. In this study, three rFIP-nha glycan mutants (N5A, N39A, N5+39A) were recombinantly expressed in *Pichia pastoris*. To test the impact on FIP-nha’s immunomodulatory activity, the phagocytotic activity, cytokine secretion, and gene expression of THP-1 macrophages were investigated. rFIP-nha and its mutants reduced macrophage phagocytosis, and induced IL-1β, IL-12 and IL-10 cytokine secretion significantly, indicating that the protein confers a pro-inflammatory behaviour on THP-1 macrophages. However, there were no obvious differences among the different glycan mutants, indicating that the observed activation mechanisms are likely glycosylation-independent. Furthermore, to study the immunomodulatory mechanism, four kinds of inflammasomes (NLRP1, NLRP3, NLRC4 and AIM2) were tested at transcriptional level. AIM2 was found to be 10-fold upregulated. Then, THP1-KO-ASC cells and AIM2 related inhibitors showed that IL-1β release induced by rFIP-nha is ASC signalling pathway dependent. Taken together, these findings suggest that rFIP-nha activates THP-1 macrophages in a pro-inflammatory way by activating the AIM2 inflammasome.

## 1 Introduction

The first line of immune defence against pathogens is the innate immune system, which is mostly composed of innate immune molecules and myeloid-derived immune cells, including monocytes, macrophages, dendritic cells (DCs), and granulocytes (Medzhitov and Medzhitov, 1998). Upon infection, innate immune cells recognize pathogen-associated molecular patterns (PAMPs) and damage-associated molecular patterns (DAMPs) (Takeuchi and Akira, 2010). The recognition of PAMPs or DAMPs initiates several signalling events, and subsequently leads to the production of proinflammatory cytokines, chemokines, reactive oxygen, nitrogen species, and antimicrobial peptides, as well as enhances phagocytic activity and rapidly removes infection (Takeuchi and Akira, 2010). In addition, the adaptive immunity gets signals from the activation of the innate immune system (Mills, 2011), to eventually sustain immune homeostasis in the host.

Macrophage is a ubiquitous cellular component present in all tissues and body compartments under homeostatic physiological conditions (Gordon and Taylor, 2005; Ginhoux and Guilliams, 2016). The eponymous function of macrophages is phagocytosis (Locati et al., 2020). Pathogens are taken up and processed in macrophages, followed by inflammasome modulation, cytokine secretion and antigen presentation to collaborative cells (Germic et al., 2019).

Inflammasome activation signifies a central pillar of innate immune signalling leading to inflammation. Recognition of PAMPs or DAMPs leads to activation of intracellular protein complexes called inflammasomes (Franchi et al., 2012). The identity of the inflammasome complex is designated by the inflammasome sensor that is activated, including NLRP1, NLRP3, NLRC4, AIM2 (Pandey et al., 2021), and their specific protein adaptors. The activated inflammasome complex includes the cysteine protease caspase-1. Caspase-1 drives the proteolytic cleavage of proinflammatory cytokines pro-interleukin (IL)-1β and IL-18, rendering these cytokines biologically active (Zaki et al., 2010). Caspase-1 also induces the proteolytic cleavage of the pore-forming protein gasdermin D (GSDMD), leading to the generation of an active N terminal domain that oligomerizes to form pores in the plasma membrane (Liu et al., 2021). These pores provide channels for the secretion of IL-1β and IL-18. Of note, short term inflammasome activation and inflammation helps to maintain the immune homeostasis, while chronic inflammation and long term inflammasome activation might damage the healthy tissue or cause tumour formation (Swanson et al., 2019; Li Y. et al., 2021; Hamarsheh and Zeiser, 2020). Therefore, it is essential to thoroughly investigate the specific mechanisms involved in each case to better understand their implications.

Pro-inflammatory responses and immune cell activation can be triggered by external stimuli, such as pathogens or exogenous compounds. Fungal immunomodulatory proteins (FIPs) are a group of small proteins with notable immunomodulatory activity. However, there is limited previous research on how FIPs activate immune cells and via which (inflammasome) mechanisms. FIP-glu (*Ganoderma lucidum*) has been described to activate the PI3K/Akt and MAPK signalling pathways after internalization by RAW264.7 macrophages (Li et al., 2019), and added N-glycosylation could enhance its anti-inflammatory activity via inhibition of p38 mitogen-activated protein kinase (MAPK) phosphorylation (Li Q.-Z. et al., 2021). FIP-fve (*Flammulina velutipes*) actives T-cells and mediates its effects via cytokine regulation through p38 MAPK signalling pathways, especially IFN-γ secretion (Wang et al., 2004). Besides, FIP-fve stimulates IFN-γ transcriptional expression in human peripheral blood mononuclear cells (hPBMCs) via the modulation of Ca^2+^ release and the activation of PKCα (Ou et al., 2009).

FIP-nha (*Nectria haematococca*) exerts considerable anti-tumour activity, with two glycosylation sites at positions N5 and N39 (Liu et al., 2022; Li et al., 2014; Bastiaan-Net et al., 2013). Earlier studies showed that FIP-nha induced IL-2 secretion on murine splenocytes (Li et al., 2014); FIP-nha upregulated IL-1β, TNF-α, IL-8 and IL-10 on THP-1 macrophage in a dose-dependent manner (Bastiaan-Net et al., 2013). However, there is little investigation on FIP-nha’s immunomodulatory activity and the underlying mechanism. Besides, the impact of glycosylation on FIP-nha’s immunomodulatory activity remains unclear. In this study, we investigated FIP-nha’s subcellular localization on THP-1 macrophages, analysed their bioactivity and further checked the related inflammasome activation. The insights into the immunomodulatory mechanism of FIP-nha is valuable, as it leads to a better understanding on FIP-nha’s bioactivity, and lays the foundation for its further utilization as an immunomodulatory therapeutic adjuvant in, for instance, cancer treatment.

## 2 Materials and methods

### 2.1 Protein preparation

The gene encoding FIP-nha (GeneBank: XM3043608) and mutations (for which the asparagine on either position 5 (N5A), position 39 (N39A) or both (N5+39A) was replaced by an alanine) were synthesized in the laboratories of BaseClear B.V. (Leiden, the Netherlands). For protein expression, the plasmid pPICZα A (Invitrogen, California, United States) was transformed into *Pichia pastoris* strain X-33 (Biolab, Beijing, China). Mutagenesis construction of FIP-nha, the induction of protein expression, and protein purification are described in a previous publication (Liu et al., 2023).

FITC (ThermoFisher, California, United States) was dissolved in DMSO with the same concentration as rFIP-nha. Protein in PBS mixed with FITC in DMSO with 10:1 ratio, and keep in 4°C overnight. FITC-labelled rFIP-nha (FITC-rFIP-nha) were separated via NAP-5 column with DPBS buffer according to its manufacturer’s protocol (GE Healthcare, Chicago, United States).

Protein concentrations (in 10 mM PBS solution; pH 7.4) were quantified by BCA Protein Assay Kit (ThermoFisher, California, United States), and all samples were snap frozen by liquid nitrogen and stored at −80°C. LPS content of rFIP-nha was tested via Endonext Kit according to the manufacturer’s protocol (Biomerieux, Marcy-l'Étoile, France). Protein information regarding concentration and LPS content is listed in Supplementary Table S1; The purified proteins visualised on SDS-PAGE gel is shown in Supplementary Figure S1.

### 2.2 Macrophage differentiation

THP-1 monocytes (ATCC (#TIB-202), Massachusetts, United States) were cultured as described previously (Hoppenbrouwers et al., 2022). For differentiation into THP-1 macrophages, cells were plated at 1 × 10^6^ cells/mL in 12-well tissue culture plates (Greiner Bio-one; 665,180) in RPMI-1640 containing 25 mM HEPES and 2 mM Glutamax (Lonza, Basel, Switzerland), supplemented with 10% FBS and 1% penicillin and streptomycin (Pen/Strep; Sigma-Aldrich, St. Louis, MO, United States) with 100 ng/mL PMA (Sigma-Aldrich, Zwijndrecht, Netherlands). After 48 h, cells were washed twice with complete RPMI medium and left for 5 days without touching the plates.

### 2.3 Localization of rFIP-nha in macrophages

Macrophages were treated with 1 μM FITC-rFIP-nha for 5 min, 10 min, 30 min, and 60 min with RPMI without phenol red. After treatment, macrophages were washed with DPBS buffer (Sigma, Zwijndrecht, Netherlands) twice and stained with 10 mM Hoechst in DPBS buffer for 10 min. Subsequently, macrophages were washed with DPBS buffer twice, and any residual FITC-dye was quenched using 0.004% trypan blue, to eliminate any signal from FITC-rFIP-nha possibly still bound to the outside of the cell. Fluorescence images were taken using the Evos FL Auto 2 (Invitrogen, California, United States) at GFP, DAPI and Trans channels with 60 times magnification separately.

### 2.4 Phagocytosis assay

Macrophages were treated with 10 μM rFIP-nha for 16 h, with the LPS amount of rFIP-nha (Supplementary Table S1; 3.15 EU/mL) as LPS-negative control (LPS-CN), and 1 μg/mL LPS as LPS-positive control (LPS + CN). Medium-exposed cells were included as non-treated control (CN). The supernatant was collected for Enzyme-Linked Immunosorbent Assay (ELISA), while the cells were further tested via 7AAD cell viability assay and phagocytosis assay. Cytotoxicity of rFIP-nha exposure to cells was analysed using the 7AAD staining (BD Biosciences, Drachten, The Nethrlands) according to the manufacturer’s protocol.

After supernatant removal, 1 mL medium +4 μg/mL AlexaFluor-conjugated *Escherichia coli* (K-12 strain) BioParticles® (Molecular Probes, Life Technologies, Eugene, OR, United States) was added to each well. Incubation occurred for 1 h in a 37 °C incubator with 5% CO_2_, followed by two washes using 1 mL of DPBS (Sigma, Zwijndrecht, Netherlands). Macrophages were harvested using 0.3 mL 0.25% Trypsin/EDTA per well, washed, resuspended with DPBS, and quantified using a CytoFlex flow cytometer (Beckman Coulter, Brea, United States) in the FITC-A channel, with data collected for up to 5000 events.

### 2.5 Activation of inflammasome expression

To investigate whether the effects of rFIP-nha were initiated by the protein itself, the rFIP-nha WT was dissolved in medium and heat-inactivated at 100°C for 10 min, and subsequently used alongside the non-heated rFIP-nha WT to analyse mRNA expression of inflammasome genes using qPCR. 2 μg/mL poly (dA:dT) (InvivoGene, California, United States) was transferred to THP-1 macrophages via Lipofectamine™ 2000 (Thermo Fisher Scientific, California, United States) following the manufacturer’s protocol as AIM2 positive control (AIM2+). 1 μg/mL Ultrapure flagellin from *S. typhimurium* (InvivoGen, California, United States) was transferred to THP-1 macrophage via Lipofectamine™ 2000 following the manufacturer’s protocol as NLRC4 positive control (NLRC4+). 1 μg/mL LPS with 5 mM ATP was considered as NLRP3 positive control (NLRP3+). Medium-treated cells were considered as non-treated control (CN). THP-1 macrophages were treated with samples for 6 h in a 37°C cell culture stove with 5% CO_2_, then media were removed, and cells were collected for RNA extraction.

### 2.6 AIM2 inhibition

ASC (apoptosis-associated speck-like protein containing a CARD domain) is an essential protein adaptor implicated in canonical inflammasome responses involving NLRP3 and AIM2 (Mathur et al., 2018). To verify the involvement of ASC, ASC knockout human monocytes (THP1-KO-ASC cells) were obtained from InvivoGen (InvivoGen, California, United States), and cultured according to the manufacturer’s protocol. For differentiation into THP1-KO-ASC macrophages, cells were plated at 1 × 10^6^ cells/mL with 1mL/well in 12-well tissue culture plates (Greiner Bio-one; 665,180) in RPMI-1640 with HEPES and Glutamax (Lonza, Basel, Switzerland) supplemented with 10% FBS, 100 ng/mL PMA (Sigma-Aldrich, Zwijndrecht, Netherlands). 1% Normocin (InvivoGen, California, United States), a formulation of three antibiotics to prevent contamination from mycoplasmas, bacteria, and fungi was used instead of 1% Pen/Strep, as advised by the HP1-KO-ASC culture instruction manual. After 48 h, cells were washed twice with complete RPMI medium and rested for 5 days without touching the plates.

The antagonists ODN TTAGGG (A151; InvivoGen, California, United States) and NLRP3/AIM2-IN-3 (J114; MedChemExpress, New York, United States) were added to THP-1 derived macrophages 1.5 h before stimulation. Both THP-1 derived and the THP1-KO-ASC macrophages were treated with 10 μM rFIP-nha (WT) for 16 h, with the LPS amount of rFIP-nha (WT) as LPS-CN, and 1 μg/mL LPS as LPS + CN. 2 μg/mL poly (dA:dT) (InvivoGen, California, United States) was transferred to macrophages via Lipofectamine™ 2000 following the manufacturer’s protocol as AIM2 positive control (AIM2+). Cells exposed to medium were included as non-treated control (CN). Medium was collected for further analysis.

### 2.7 ELISA

In the macrophage supernatant, IL-1β, IL-12/IL-23 (p40) (shown as IL-12 in the following content) and IL-10 were measured according to the manufacturer’s protocol (BioLegend, Koblenz, Germany). All samples were measured using a Tecan Infinite 200PRO (Tecan, Männedorf, Switzerland).

### 2.8 RNA extraction and cDNA synthesis

Macrophages were treated with 10 μM rFIP-nha for 3 h and 6 h, with the LPS amount of rFIP-nha as LPS-CN (Supplementary Table S1; 3.15 EU/mL), and 1 μg/mL LPS as LPS + CN. RNA was extracted by lysing cells with 400 μL TRIzol (Invitrogen, Bleiswijk, Netherlands) for each well. This was followed by RNeasy (Qiagen, Venlo, Netherlands) on column clean-up according to manufacturer’s protocol. The integrity of the ribosomal RNA was verified through 1% agarose (Eurogentec, Liège, Belgium) gel electrophoresis. RNA concentration and purity were checked using the Nanodrop spectrophotometer system (Nanodrop Technologies, Wilmington, DE, United States) and only samples with a ratio (Abs 260/280 nm) between 1.8 and 2.1 were used for qPCR. Subsequently, cDNA was synthesized using iScript (Bio-Rad, Veenendaal, Netherlands) and 200 ng RNA per reaction, according to manufacturer’s protocol.

### 2.9 qPCR for inflammasome activation

qPCR was performed as previously described by Tang et al. (2017). Primers were derived from the Harvard Primerbank (http://pga.mgh.harvard.edu/primerbank/) and synthesized by Biolegio (Nijmegen, Netherlands). The cycling conditions used for amplifying the target sequences using the CFX96 Touch Real-Time PCR Detection System (Bio-Rad) were as follows: 90 s at 95°C, followed by 40 cycles at 95°C for 10 s, 58°C for 10 s and 72°C for 15 s, with a final elongation step at 72°C for 2 min (Tang et al., 2017). qPCR was performed in technical duplicate, and all samples were normalized to the geometric means of RPLP0 (ribosomal protein lateral stalk subunit P0; 60S acidic ribosomal protein P0) and ActinB expression (ΔCT normalisation towards two reference genes) and then normalised against medium-stimulated macrophages (ΔΔCT normalisation, setting NT to 1) via the ΔΔCT method using the qbase + software (Biogazelle, Gent, Belgium). The primers’ information is listed in Supplementary Table S2.

### 2.10 Statistics

Phagocytosis assays and cytokine secretion assays were independently repeated five times (n = 5 independent biological replicates); other experiments were repeated three times independently (n = 3). Statistical analyses were carried out using Graphpad Prism 8. All parameters are presented as means ± SEM. A One-way ANOVA with a Dunnett post-hoc test to correct for multiple comparisons was used to assess the parameters for significance (p < 0.05; p < 0.1 is considered a trend). All groups were only compared to the non-treated control (NT). Outliers were identified using the ROUT method with Q = 1%. Graphs were plotted using Graphpad Prism 8.

## 3 Results

### 3.1 rFIP-nha uptake by macrophages

**FIGURE 1:**
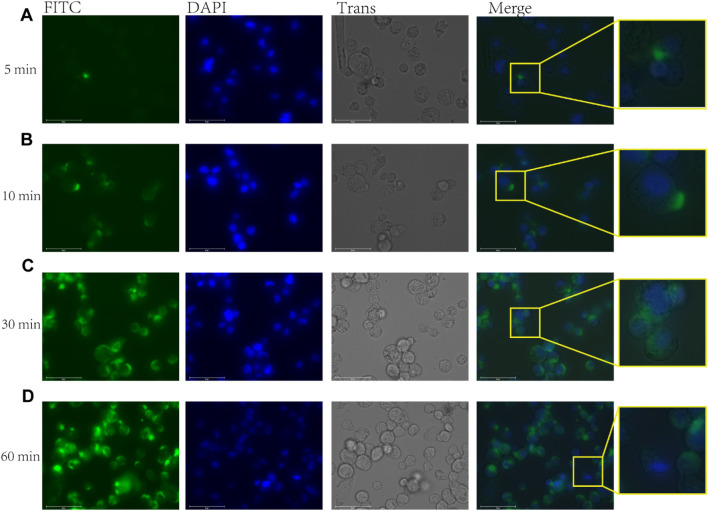
FITC-rFIP-nha taken up by THP-1 macrophages, with FITC-rFIP-nha shown on FITC channel (green), Hoechst stained nuclear acids shown on DAPI channel (blue), and cell image shown on Trans channel. The FITC-labelled rFIP-nha that was not taken up from the media was quenched using trypan blue. **(A)** FITC-rFIP-nha taken up by THP-1 macrophages in 5 min; **(B)** 10 min; **(C)** 30 min; **(D)** 60 min.

To investigate the immunomodulatory activity of rFIP-nha on macrophages, we first determined whether the rFIP-nha was taken up by the macrophages using FITC-rFIP-nha (no cytotoxicity was observed on macrophages in the tested concentration range up to 1 µM FIP-nha; data not shown). After application, the macrophages started gradually to take up FITC-rFIP-nha (Figure 1). Within 1 h, FITC-rFIP-nha was present in the cytoplasm of almost all macrophages (see merged enlargements in Figures 1A–D). These results suggest that rFIP-nha could exert its activity inside the cell.

### 3.2 rFIP-nha decreased macrophage phagocytosis

**FIGURE 2:**
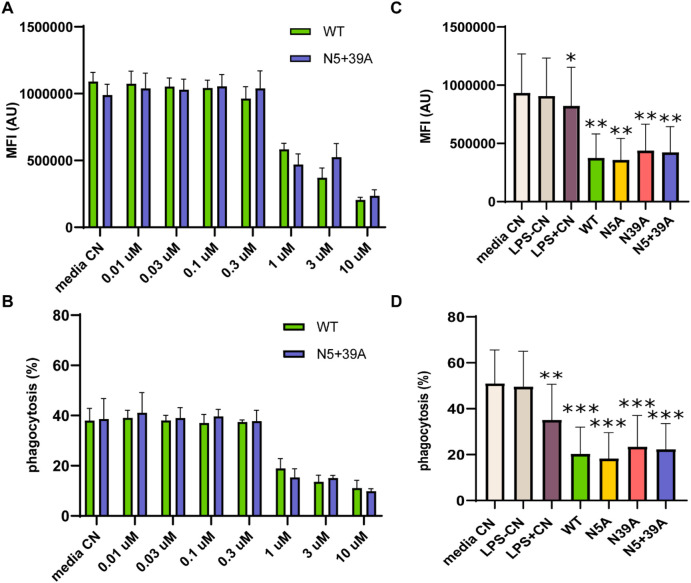
rFIP-nha decreases THP-1 macrophage phagocytosis of fluorescently labelled *Escherichia coli*. **(A)** the amount of *Escherichia coli* particles consumed by each THP-1 macrophage after treatment with WT or N5+39A for 16 h in a concentration range from 0.01 µM to 10 μM, measured as MFI; **(B)** the percentage of THP-1 macrophages that phagocytosed *Escherichia coli* particles after treatment with WT or N5+39A for 16 h in a concentration range from 0.01 µM to 10 μM; **(C)** the amount of *Escherichia coli* particles phagocytosed by each THP-1 macrophage treated with 10 μM WT, N5A, N39A or N5+39A for 16 h, measured as MFI; **(D)** the percentage of THP-1 macrophages that phagocytosed *Escherichia coli* particles after treatment with 10 μM WT, N5A, N39A or N5+39A for 16 h. The highest LPS content of rFIP-nha and its mutants was considered as LPS-CN; 1 μg/mL LPS was used as LPS + CN; medium was used as non-treated control (Media CN). All values were compared to media CN, with *p < 0.1, ** p < 0.01, *** p < 0.001, ****p < 0.0001.

Macrophages are crucial in the elimination of diseased and damaged cells through phagocytosis. To assess the impact of rFIP-nha on macrophage phagocytic activity, we considered both the percentage of macrophages engaged in the phagocytosis of fluorescent *Escherichia coli* particles and the quantity of *E. coli* particles consumed in each macrophage, measured as mean fluorescence intensity (MFI). As shown in Figure 2, rFIP-nha dose-dependently inhibited macrophage phagocytosis. Initially, the MFI of macrophages was around 1,000,000, with a phagocytic rate of approximately 40% in the control media treatment. Exposure to rFIP-nha (0.01 μM–10 μM) led to a gradual decrease in macrophage MFI to approximately 200,000 and a reduction in phagocytosis to 10% (Figures 2A,B; Supplementary Figures S3–S5). As pro-inflammatory macrophages (M1) have been described to be less phagocytotic compared with anti-inflammatory (M2) macrophages (Hoppenbrouwers et al., 2022), this could potentially indicate that rFIP-nha might skew macrophages towards a pro-inflammatory phenotype. Meanwhile, rFIP-nha glycosylation did not seem to affect phagocytosis (Figures 2C,D; Supplementary Figures S6–S8). A degree of cell aggregation was observed for cells exposed to rFIP-nha and its mutants (Supplementary Figure S9). This is likely caused by cross-linking of cell surface carbohydrates, often observed by lectin-like proteins (Chen et al., 2021).

### 3.3 rFIP-nha enhanced pro-inflammatory cytokine secretion

**FIGURE 3:**
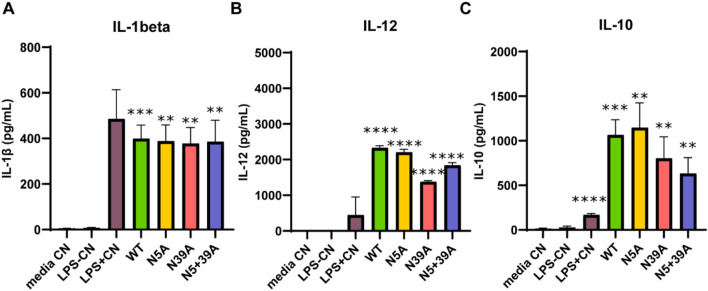
Cytokine secretion of THP-1 macrophages treated with rFIP-nha and its mutants. The highest LPS content of rFIP-nha and its mutants was added as LPS-CN; 1 μg/mL LPS was added as LPS + CN (M1); media CN were exposed to just medium; macrophages were exposed to 10 μM of rFIP-nha and its mutants for 16 h. **(A)** IL-1β secretion of THP-1 macrophages treated with rFIP-nha and its mutants; **(B)** IL-12 secretion of THP-1 macrophages treated with rFIP-nha and its mutants; **(C)** IL-10 secretion of THP-1 macrophages treated with rFIP-nha and its mutants. All values were compared to media CN, with ** p < 0.01, *** p < 0.001, ****p < 0.0001.

To further investigate the rFIP-nha’s effect on macrophage cytokine secretion, IL-1β, IL-12 and IL-10 were measured. As shown in Figure 3, rFIP-nha increased the production of all cytokines significantly compared to the medium control and LPS negative control. Interestingly, the secretion of IL-12 and IL-10 induced by rFIP-nha was significantly higher compared to the LPS positive control. These results indicate that rFIP-nha induces a pro-inflammatory THP-1 macrophage phenotype, which is similar, but not exactly like M1 macrophages induced by LPS. There was no obvious difference between the WT rFIP-nha and the glycosylation mutants in IL-1β secretion. Noteworthy, N39A induced less IL-12 and IL-10 secretion compared with other variants (Supplementary Figure S1). From microscopic photo analysis (Supplementary Figure S2), it was concluded that no obvious morphological changes were observed after exposure to rFIP-nha.

### 3.4 rFIP-nha modulated inflammasome activation

**FIGURE 4:**
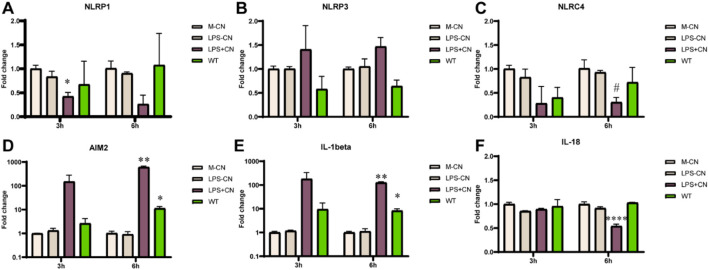
THP-1 macrophage gene expression of inflammasomes, IL-1β and IL-18 after rFIP-nha (WT) treatment. The LPS content of rFIP-nha was added as LPS-CN; 1 μg/mL LPS was added as LPS + CN (M1); Medium-treatment was included as non-treated control (M-CN). THP-1 macrophages were exposed to 10 μM of rFIP-nha for 3 h and 6 h. **(A)** NLRP1 gene expression of THP-1 macrophages; **(B)** NLRP3 gene expression of THP-1 macrophages; **(C)** NLRC4 gene expression of THP-1 macrophages; **(D)** AIM2 gene expression of THP-1 macrophages; **(E)** IL-1β gene expression of THP-1 macrophages; **(F)** IL-18 gene expression of THP-1 macrophages. All values were compared to M-CN, with #p < 0.1, * p < 0.05, ** p < 0.01, ****p < 0.0001.

To further investigate the mechanism behind the secretion of IL-1β by rFIP-nha treated macrophages, the main classes of inflammasomes were evaluated at transcriptional level. As no significant difference of IL-1β secretion was observed between rFIP-nha and its mutants, we used rFIP-nha WT as a representative in the next experiments. As shown in Figure 4, the AIM2 inflammasome and IL-1β were significantly up-regulated after rFIP-nha treatment for 6 h. IL-1β and AIM2 expression increased around 10-fold, indicating that rFIP-nha induced IL-1β expression and secretion via AIM2 inflammasome regulation.

**FIGURE 5:**
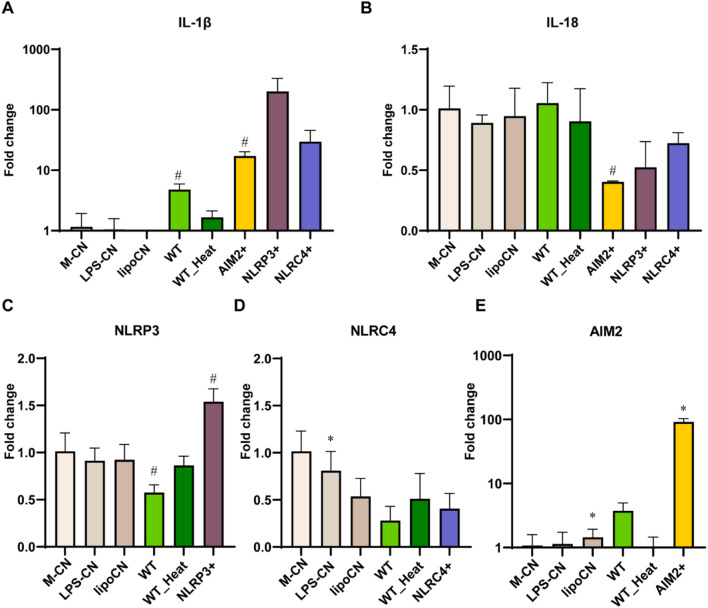
THP-1 macrophages gene expression of inflammasomes **(A)** IL-1β, **(B)** IL-18, **(C)** NLRP3, **(D)** NLRC4, **(E)** AIM2. IL-1β and IL-18 after 10 μM rFIP-nha (WT), heated WT (WT_Heat), and inflammasomes positive control treatment for 6 h. The LPS content of rFIP-nha was added as LPS-CN; Lipofectamine™ 2000 was added as lipoCN; 1 μg/mL LPS with 5 mM ATP was considered as NLRP3 positive control (NLRP3+); 1 μg/mL Ultrapure flagellin from *S. typhimurium* was transferred to THP-1 macrophages via Lipofectamine™ 2000 as NLRC4 positive control (NLRC4+); 2 μg/mL poly (dA:dT) was transferred to THP-1 macrophages via Lipofectamine™ 2000 as AIM2 positive control (AIM2+); Non-treated cells were exposed to medium (M-CN); WT was heated at 100°C for 10 min and diluted in the medium as WT_Heat. All values were compared to M-CN, with #p < 0.1, *p < 0.05.

As rFIP-nha was expressed by *P. pastoris*, we investigated whether (a non-protein) yeast contamination could induce the false appearance. We inactivated rFIP-nha with heat treatment. The heated rFIP-nha failed to modulate inflammasome activation (Figure 5), suggesting that the activation of the AIM2 inflammasome was induced by rFIP-nha rather than by (for instance) DNA contamination originating from yeast. To validate inflammasome modulation, positive controls for activation of AIM2, NLRP3, and NLRC4 were included. Although not statistically significant, the AIM2 positive control (p = 0.06) and WT (p = 0.08) upregulated IL-1β at transcriptional level (Figure 5A), but not in IL-18 (Figure 5B). The AIM2 and NLRP3 (not significant) positive controls also upregulated their respective inflammasomes at the transcriptional level (Figures 5C,E). RFIP-nha WT showed a trend in *AIM2* upregulation (p = 0.06). However, the NLRC4 positive control did not upregulate NLRC4 expression. It may be necessary to optimize the timing of gene expression assessment for this particular inflammasome complex.

**FIGURE 6:**
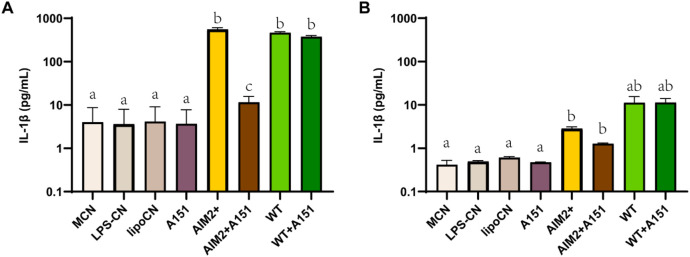
IL-1β cytokine secretion of THP-1 macrophages **(A)** and THP1-KO-ASC macrophages **(B)** treated with AIM2 positive control, rFIP-nha WT and inhibitor A151 (n = 3). The LPS content of rFIP-nha was added as LPS-CN; Lipofectamine™ 2000 was added as lipoCN; 2 μg/mL poly (dA:dT) was transferred macrophages via Lipofectamine™ 2000 as AIM2 positive control (AIM2+); cells exposed to medium we included as non-treated control (M-CN). Macrophages were treated with samples for 16 h. Group “a,” group “b” and group “c” are with significant difference (p < 0.05), group “ab” shows no significant difference between group “a” and “b.”

To investigate whether IL-1β release is indeed AIM2 inflammasome-dependent, a THP1-KO-ASC macrophage cell line and two inhibitors associated with AIM2 inflammasome regulation were tested. The apoptosis-associated speck-like protein (ASC) is an essential adaptor of the inflammasomes AIM2 and NLRP3 to process proinflammatory cytokines such as IL-1β (Li Y. et al., 2021). The IL-1β release induced by rFIP-nha WT from ASC-KO-THP-1 macrophages was significantly lower than from native THP-1 macrophages, indicating that the observed IL-1β release is at least partially ASC pathway dependent (Figure 6). A151, a synthetic oligodeoxynucleotide, functions as a competitive inhibitor of the dsDNA–AIM2 interaction (Kaminski et al., 2013). It did not downregulate the IL-1β release induced by rFIP-nha WT, but partially blocked the inflammasome assembly in both macrophages. In the THP1 macrophages the A151 contributes to a more than 500 pg/mL downregulation (P value around 0.01), while it also diminishes IL-1β release in the ASC-KO-THP-1 macrophage (P value around 0.05). J114, another inhibitor, disrupts the interaction of NLRP3 or AIM2 with the adaptor protein ASC and inhibits ASC oligomerization to diminish IL-1β release (Jiao et al., 2022). This inhibitor worked less effectively in inhibiting the effects of the AIM2 positive control, while exhibiting limited efficacy in IL-1β release induced by rFIP-nha (Supplementary Figure S10). This discrepancy may arise from the possibility that rFIP-nha interacts with the AIM2 inflammasome in a manner distinct from dsDNA. Also, there might be other ASC-related pathways involved, as the IL-1β release induced by rFIP-nha in the THP1-KO-ASC macrophages was still 2-fold higher than induced by the AIM2 positive control.

## 4 Discussion

In this study, we investigated the immunomodulatory properties of rFIP-nha on THP-1 macrophages, with a particular focus on elucidating the underlying mechanism. Additionally, we sought to understand how glycosylation influences the immunomodulatory activity of rFIP-nha. Our findings indicate that rFIP-nha elicits a pro-inflammatory response in THP-1 macrophages through the activation of the AIM2 inflammasome. Furthermore, we observed that glycosylation has an impact on the release of IL-12 and IL-10 induced by rFIP-nha, but it does not affect the release of IL-1β or the reduction of phagocytosis.

Macrophages are innate immune cells that are found throughout the body in both tissue-resident and monocyte-derived forms (Gordon and Taylor, 2005; Davies et al., 2013). They guard their host against unwanted substances (Franken et al., 2016), and remove them via phagocytosis. The range of substances cleared by macrophage phagocytosis is wide and known to include dead or dying host cells, antibody-labelled cells and specific pathogens (Gordon, 2016; Joffe et al., 2020). For instance, macrophages could take up FITC-conjugated bacteria and this effect could be enhanced using ginsenoside, leading to an anti-inflammatory response in primary macrophages from mice (Xin et al., 2019). LL-37 antimicrobial peptides promote macrophage uptake of neutrophil extracellular traps (NETs) which could modulate LPS-induced cytokine responses in human primary macrophages (Lazzaretto and Fadeel, 2019). These findings are consistent with our results, as rFIP-nha was quickly taken up by macrophages (Figure 1), leading to the downstream reactions.

Phagocytosis is not only a fundamental function of macrophages, but also a distinguished factor among different macrophage phenotypes. Depending on the activating signals, macrophages can be categorized into two types: classically activated macrophages (M1) and alternatively activated macrophages (M2) (Locati et al., 2020; Zhu et al., 2015; Murray, 2017). M1 macrophages release pro-inflammatory cytokines such as TNF-α, IL-1β, and IL-12, chemokines like CCL-2 and CXCL-10, and nitric oxide (NO); whereas M2 macrophages produce higher levels of anti-inflammatory cytokines, such as IL-10 and TGF-β1 (Wang et al., 2021; Boutilier and Elsawa, 2021; Liu et al., 2014). In both THP-1 macrophages and primary macrophages, M2 macrophages exhibit a higher capacity for phagocytosis compared to M1 macrophages (Hoppenbrouwers et al., 2022; Xin et al., 2019). As rFIP-nha lowered macrophage phagocytosis for both the percentage of cells engaged in phagocytosis as well as the number of particles absorbed during phagocytosis, without causing cytotoxic effects, (Figure 2), it likely inhibits the intrinsic phagocytosis activity and switch the macrophage phenotype to a pro-inflammatory direction, similar as to what was observed for the LPS + CN control. Pro-inflammatory macrophages typically secrete cytokines as IL-1β, IL-12 and TNF-α (Wang et al., 2021; Boutilier and Elsawa, 2021). Furthermore, A higher IL-12/IL-10 ratio is always observed in M1 macrophages (Hoppenbrouwers et al., 2022; Mantovani et al., 2005). rFIP-nha induced more IL-12 and IL-10 secretion by macrophages (Figure 3), leading to a higher IL-12/IL-10 ratio compared to the medium control. In addition to the previously mentioned phagocytosis, the increased pro-inflammatory cytokine secretion induced by rFIP-nha provides further evidence of macrophage reprogramming.

Previously, other FIPs have also been described to have pro-inflammatory effects on macrophages. rFIP-SJ75, which is a chimera composed of LZ-8, FIP-fve and FIP-vvo, triggered RAW264.7 macrophages by promoting M1 polarization and initiating pro-inflammatory responses (Shao et al., 2019). Immunomodulatory protein from *Poria cocos* (PCP) can stimulate RAW 264.7 macrophages *in vitro* through the induction of TNF-α and IL-1β as well as the regulation of nuclear factor-kappa B (NF-κB)-related gene expression (Chang et al., 2009). A study performed on crude protein from 13 kinds of edible mushrooms found that edible mushroom proteins promote the polarisation of macrophages into classical M1-type macrophages, as indicated by cytokine secretion (Xu et al., 2022). Of note, the LPS content should be always taken into consideration as it could cause pro-inflammatory response from macrophages, thereby leading to false positive results.

IL-1β is a pro-inflammatory cytokine and its maturation depends on the activation of caspase-1, while caspase-1 activation is triggered by inflammasome activation (Alehashemi and Goldbach-Mansky, 2020). Inflammasomes are activated in response to various stimuli (Pandey et al., 2021; Li Y. et al., 2021). Upon stimulation, a cytosolic multiprotein complex forms, consisting of the innate immune receptor protein, adapter protein ASC, and the inflammatory protease caspase-1. Inflammasomes can be categorized into different types based on their composition, including NLRP1, NLRP3, NLRC4, AIM2, and others (Pandey et al., 2021). We found that rFIP-nha activated macrophages through the AIM2 inflammasome (Figures 4–6). Most likely rFIP-nha is taken up by macrophages (Figure 1), after which rFIP-nha upregulates IL-1β and AIM2 inflammasome gene expression (Figure 4), activating a caspase activity cascade that leads to the induction of IL-1β, IL-12 and IL-10 secretion (Figure 3). *AIM2* upregulation by rFIP-nha was in line with observations for *Mycobacterium bovis*, the causative agent of bovine tuberculosis, which triggered interleukin 1β (IL-1β) in J774A.1 macrophages via the AIM2 inflammasome (Yang, 2013). Upregulation of relative *AIM2* gene expression was most pronounced at 24 h postinfection and approximately 6-fold compared to non-infected macrophages. In addition, a similar (approx. 4-fold) upregulation of *AIM2* in M2-polarised THP-1 macrophages was seen for the plant *Withania somnifera* derived lactone triterpenoid Withaferin A (Ngoungoure and Owona, 2019).

**FIGURE 7:**
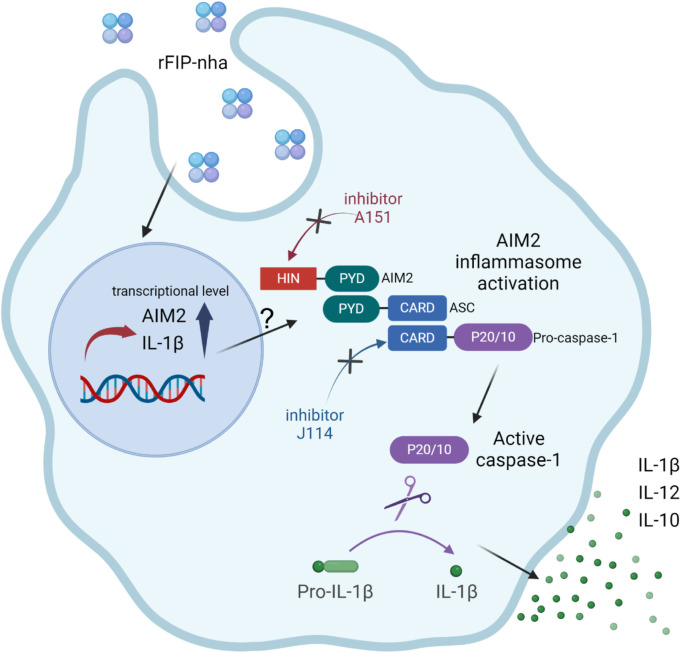
Schematic representation illustrating the pro-inflammatory effects of rFIP-nha on THP-1 macrophages.

To activate the AIM2 inflammasome, rFIP-nha should be able to translocate from the phagosome into the cytosol, either as intact protein or as partial degraded peptides that are still bioactive. Contrary to phagocytosed Gram-positive bacteria, internalised proteins do not appear to activate acidification of the phagosome to promote degradation (Sokolovska et al., 2013), suggesting that rFIP-nha might indeed translocate partly intact to the cytosol. The exact mechanism of how rFIP-nha is internalised and able to interact with AIM2 in the cytosol requires further investigation. Indeed, the involvement of the ASC-related pathway is evident (Figure 6) but exactly how rFIP-nha activates the AIM2 inflammasome pathway is not yet completely clear. The lack of a strong inhibition by the AIM2 inhibitor A151 suggests that rFIP-nha interacts with AIM2 in a different manner than dsDNA. A151 binds to the DNA-binding HIN200C domain of AIM2 in a competitive way, thereby preventing ASC recruitment via the pyrin (PYD) domain (Kaminski et al., 2013). Perhaps rFIP-nha interacts directly with the AIM2-PYD domain facilitating recruitment and oligomerization of ASC, which would explain the observed lack of inhibition by A151. Alternatively, or additionally, rFIP-nha may directly facilitate ASC oligomerization via PYD-interaction, which would be in line with the results seen for the ASC-KO cell line and the J114 inhibitor assays. Future research should reveal whether protein-complexation with AIM2 or ASC follows a different signal transduction pathway towards IL-1β release than the one activated upon DNA-AIM2 interaction. Despite some remaining uncertainties, the available evidence strongly suggests that rFIP-nha exerts an activating effect on macrophages, skewing them towards a pro-inflammatory-like phenotype through modulation of the AIM2 inflammasome (Figure 7).

Cui and co-workers (Cui et al., 2024) recently highlighted the significance of AIM2 inflammasome activation in cancer treatment, emphasizing development of effective immunotherapeutic approaches targeting AIM2 modulation. The presence of dsDNA-containing exosomes in (cancer) cells is considered an oncological biomarker and DNA release by cancer cells activates AIM2 in macrophages (Chew et al., 2023). In colorectal cancer patients, a reduced or absent AIM2 expression correlated with an up to 3-times rise in overall mortality compared to cancer patients with unaltered AIM2 expression (Dihlmann et al., 2014). Our data showed a clear rFIP-nha driven upregulation in AIM2 gene expression; in doing so rFIP-nha treated macrophages may become more sensitive in detecting cancer cell-secreted dsDNA and thus more effective in inducing cancer cell death. Follow-up research should therefore also include a deeper understanding of macrophage AIM2-triggered cancer cell death, specifically looking at pyroptosis-, apoptosis- and autophagic-driven mechanisms, to determine the effectiveness of rFIP-nha as an anti-cancer immunotherapeutic.

To conclude, in this study we produced rFIP-nha and its glycation mutants (N5A, N39A, N5+39A) in order to explore their immune modulating effects on THP-1 macrophages by investigating phagocytosis activity and cytokine secretion. The activation mechanism of secreted cytokines was studied by checking inflammasome pathways activation using dedicated gene expression, AIM2-related inhibitors and an ASC knock-out cell line. Our findings reveal that rFIP-nha activates THP-1 macrophages in a pro-inflammatory way via primarily AIM2 inflammasome activation, in addition to perhaps other inflammasomes.

## Data Availability

The datasets presented in this study can be found in online repositories. The names of the repository/repositories and accession number(s) can be found in the article/Supplementary Material.
