# Supplementary material for: rFIP-nha activates macrophages towards a pro-inflammatory phenotype via AIM2 inflammasome modulation
Source: Front Cell Dev Biol. 2025 Apr 28;13:1533742. doi: 10.3389/fcell.2025.1533742 (PMC12066430; doi:10.3389/fcell.2025.1533742)
Supplement: Supplementary file 1 [file DataSheet1.docx]

**rFIP-nha activates macrophages towards a pro-inflammatory phenotype via AIM2 inflammasome modulation**

Yusi Liu^a,b,c,^*, Zhen Li^c^, Harry Wichers^a,b^, Shanna Bastiaan-Net^a,†,^* , Tamara Hoppenbrouwers^a,d,†^

^a^ Wageningen Food and Biobased Research, Wageningen University and Research, Wageningen 6708WG, the Netherlands

^b^ Laboratory of Food Chemistry, Wageningen University, Wageningen 6708WG, the Netherlands

^c^ Laboratory of Biomanufacturing and Food Engineering, Institute of Food Science and Technology, Chinese Academy of Agriculture Sciences, Beijing 100193, China

^d^ Laboratory of Food Quality and Design, Wageningen University, Wageningen 6708WG, the Netherlands

^†^ These authors share senior authorship

* Corresponding authors.

*E-mail addresses:* [Yusiliu0226@hotmail.com](mailto:Yusiliu0226@hotmail.com) (Y. Liu), [shanna.bastiaan@wur.nl](mailto:shanna.bastiaan@wur.nl) (S. Bastiaan-Net)

**Supplementary data**

**Table S1** The protein concentration and LPS content of rFIP-nha

|  | WT | N5A | N39A | N5+39A | FITC-rFIP-nha |
| --- | --- | --- | --- | --- | --- |
| **Concentration (mg/mL)** | 20.5 | 19.1 | 11.3 | 12.9 | 3.3 |
| **Molarity (μM)** | 1521.2 | 1481.9 | 842.0 | 954.1 | 240.1 |
| **LPS (EU/mL)** | 1.39 | 0.76 | 1.37 | 3.15 | - |

**Table S2** qPCR primer information. Primer efficiency was determined using a cDNA dilution series ranging from 10^0^ to 10^-4^, in which the slope of the linear standard curve was used to calculate the primer efficiency using the equation: E = -1+10^(-1/slope)^ by the Biorad Software. A slope of -3.322 corresponds to a primer efficiency of 100%.

| Primer | Sequence 5’-3’(bp) | EST ID/Primer bank ID/reference | Working concentration (μM) | T_m_ (℃) | Primer efficiency |
| --- | --- | --- | --- | --- | --- |
| NLRP1-F | GCAGTGCTAATGCCCTGGAT | 119393880c1 | 1.6 | 62 | 120.7% |
| NLRP1-R | GAGCTTGGTAGAGGAGTGAGG | 119393880c1 | 1.6 | 61 | 120.7% |
| NLRP3-F | GAG GCA ACA CTC TCG GAG AC | [1] | 0.4 | 61 | 104.6% |
| NLRP3-R | TCT GGC TGG AGG TCA GAA GT | [1] | 0.4 | 62 | 104.6% |
| NLRC4-F | TGCATCATTGAAGGGGAATCTG | 312433959c2 | 0.4 | 60 | 118.8% |
| NLRC4-R | GATTGTGCCAGGTATATCCAGG | 312433959c2 | 0.4 | 60 | 118.8% |
| AIM2-F | TCAAGCTGAAATGAGTCCTGC | 4757733c2 | 0.4 | 60 | 115.5% |
| AIM2-R | CTTGGGTCTCAAACGTGAAGG | 4757733c2 | 0.4 | 60 | 115.5% |
| IL-1β-F | GTGGCAATGAGGATGACTTGTTC | NM_000576.2 | 0.1 | 63 | 95.1% |
| IL-1β-R | TAGTGGTGGTCGGAGATTCGTA | NM_000576.2 | 0.1 | 64 | 95.1% |
| IL-18 | AACAAACTATTTGTCGCAGGAAT | 342349317c1 | 0.4 | 55 | 98.5% |
| IL-18 | TGCCACAAAGTTGATGCAAT | 342349317c1 | 0.4 | 55 | 98.5% |
| RPLP0-F | GCAATGTTGCCAGTGTCTG | NM_001002.3 | 0.1 | 61 | 93.9% |
| RPLP0-R | GCCTTGACCTTTTCAGCAA | NM_001002.3 | 0.1 | 60 | 93.9% |
| ActinB-F | CTGGAACGGTGAAGGTGACA | NM_001101 | 0.1 | 64 | 93.7% |
| ActinB-R | AAGGGACTTCCTGTAACAATGCA | NM_001101 | 0.1 | 64 | 93.7% |


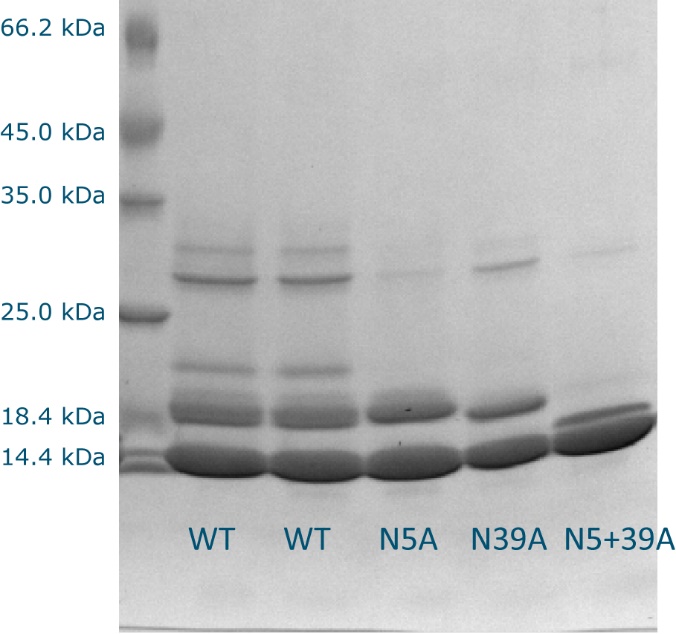


**Figure S1** WT and mutants (around 2 μmol protein loaded) shown on SDS-PAGE. Purification from the *Pichia pastoris* strain X33 was performed using Ni-TNA agarose followed by Superdex 200 size exclusion chromatography. Lane 1: Protein marker; Lane 2: rFIP-nha WT after Ni-NTA purification; Lane 3: rFIP-nha WT after size exclusion chromatography purification; Lane 4-6: rFIP-nha mutants after size exclusion chromatography purification. Multiple bands on gel indicate glycosylation variances. WT is secreted as a mixture of double glycosylated (ca. 20kDa), single glycosylated (ca. 19kDa) and non-glycosylated (ca. 14kDa). Oligomers are visible higher up the SDS PAGE gel. For more information about the glycosylation mutant, we refer to [2].


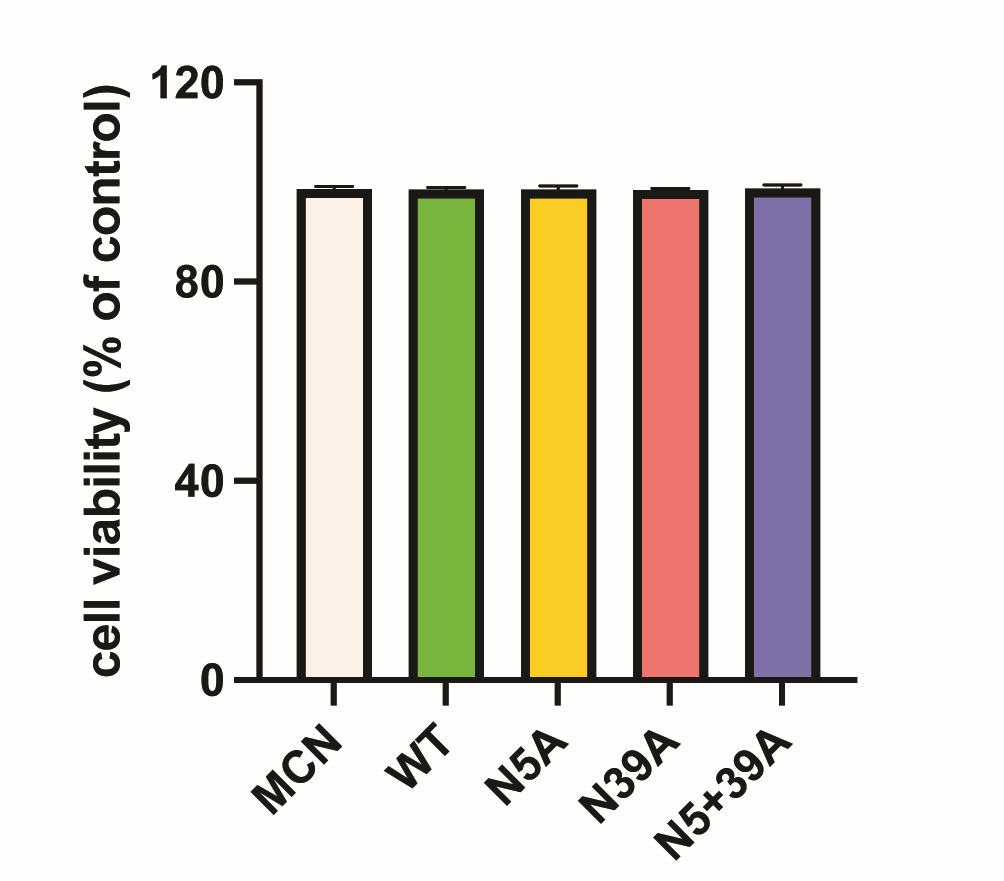


**Figure S2** Cell viability of macrophage exposed to 10 µM rFIP-nha and its glycosylation mutants for 16h as measured by 7AAD staining. Medium-treated cells were taken along as non-treated cells (M-CN).


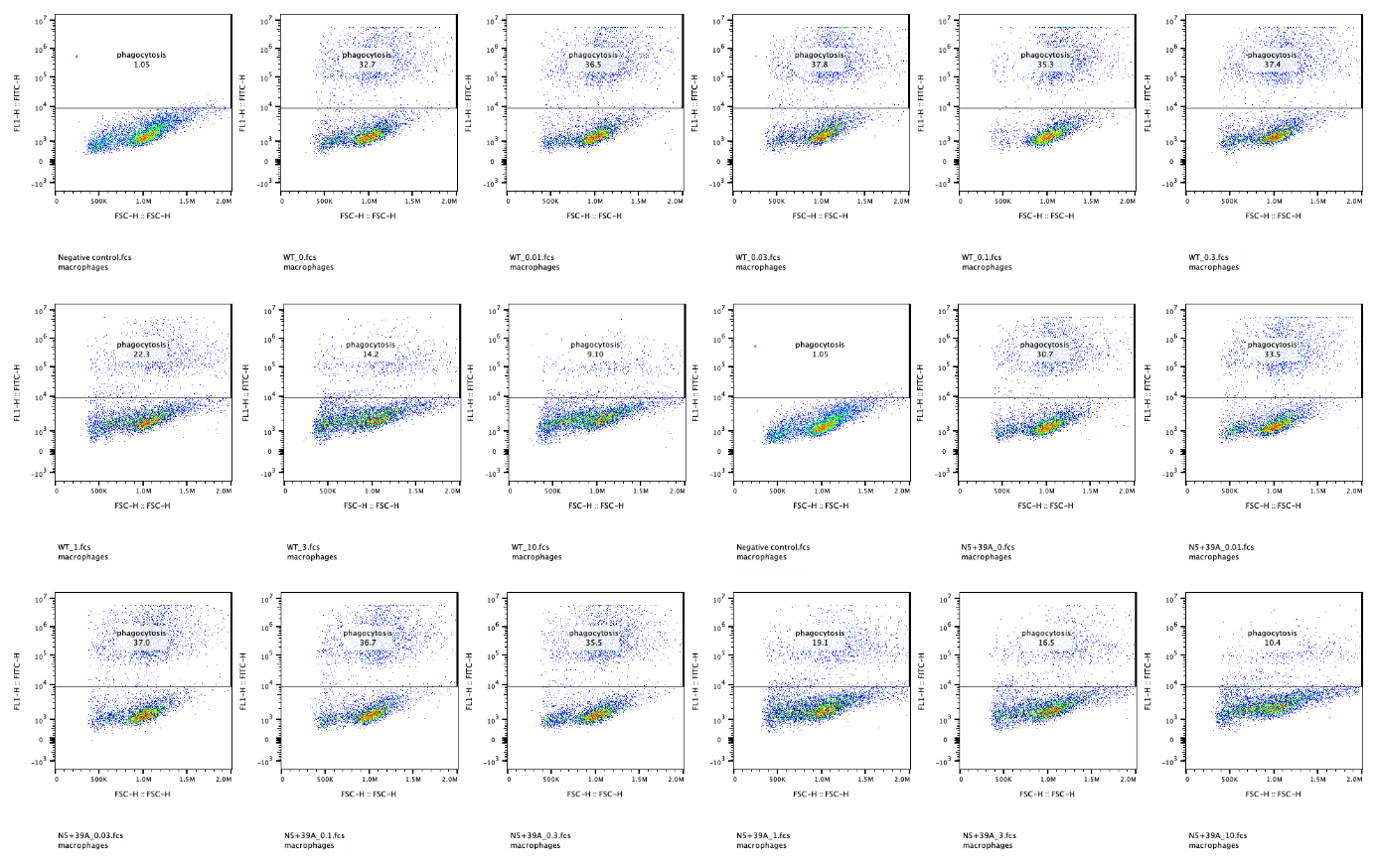


**Figure S3** rFIP-nha decreases THP-1 macrophage phagocytosis of fluorescently labelled *E. coli*. The CytoFlex flow cytometer plots display the percentage of THP-1 macrophages that have phagocytosed *E. coli* particles after exposure to rFIP-nha WT or N5+39A for 16h in a concentration range from 0.01 µM to 10 µM (n=1). Control cells were exposed to just medium.


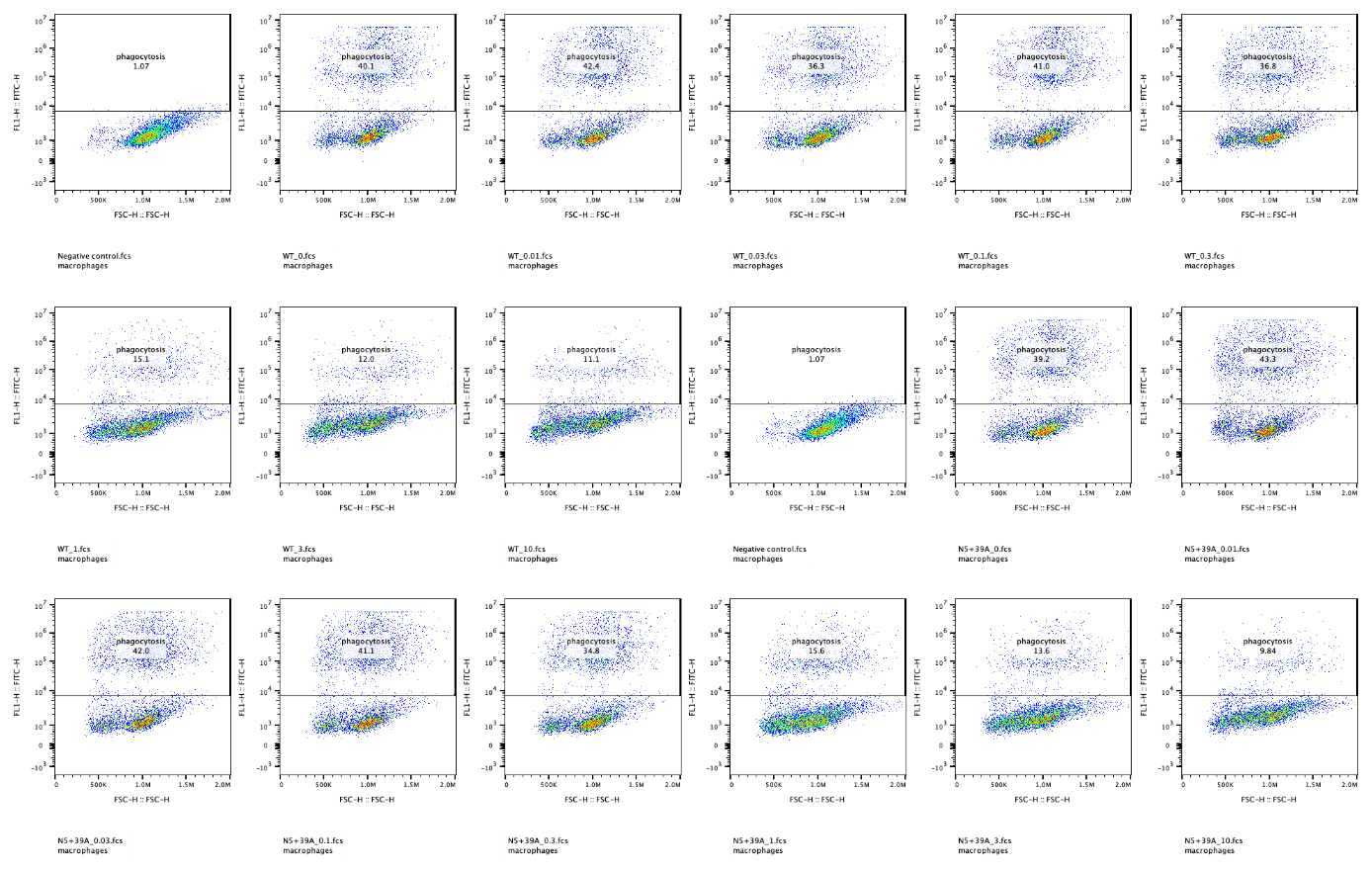


**Figure S4** rFIP-nha decreases THP-1 macrophage phagocytosis of fluorescently labelled *E. coli*. The CytoFlex flow cytometer plots display the percentage of THP-1 macrophages that have phagocytosed *E. coli* particles after exposure to rFIP-nha WT or N5+39A for 16h in a concentration range from 0.01 µM to 10 µM (n=2). Control cells were exposed to just medium.


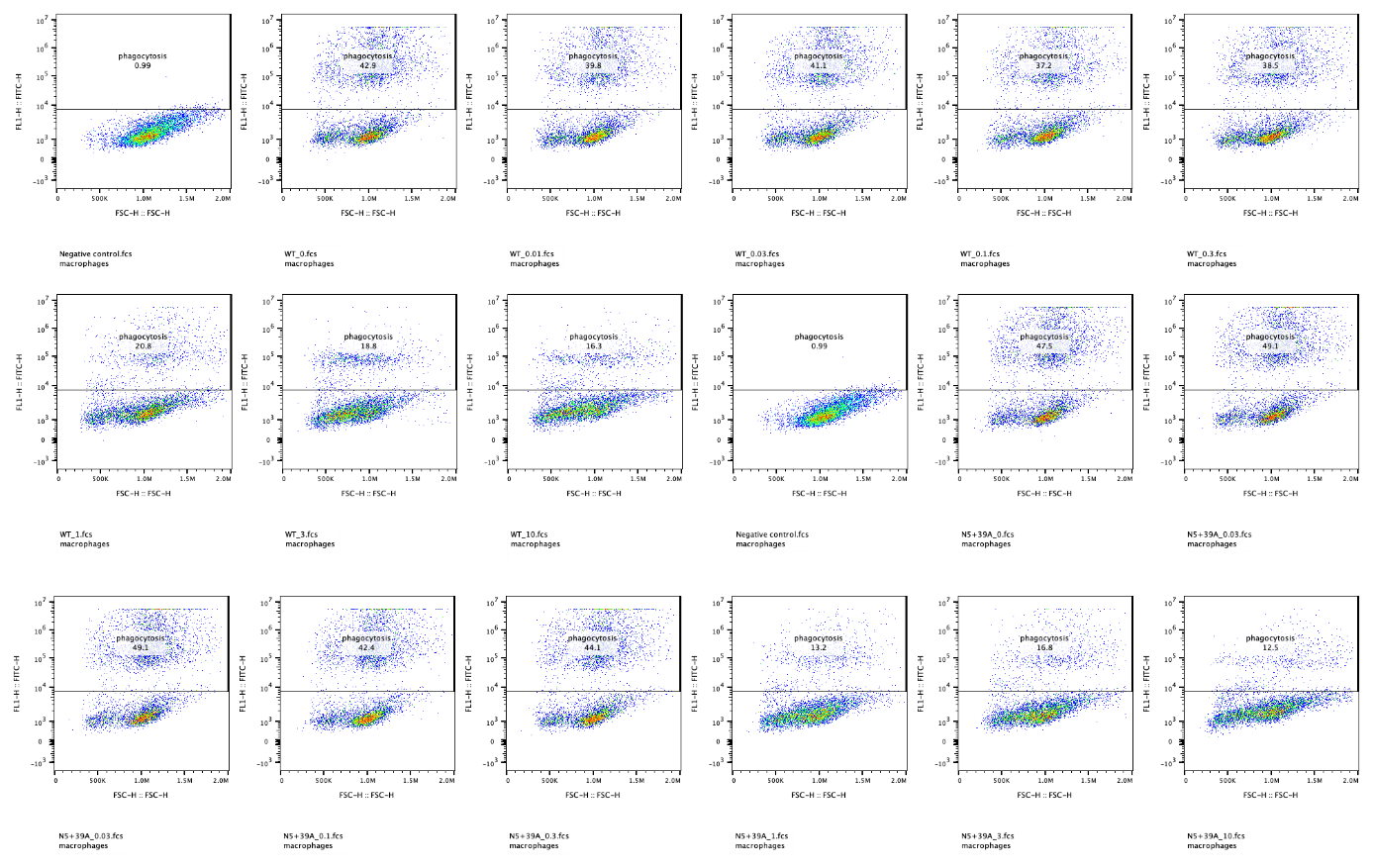


**Figure S5** rFIP-nha decreases THP-1 macrophage phagocytosis of fluorescently labelled *E. coli*. The CytoFlex flow cytometer plots display the percentage of THP-1 macrophages that have phagocytosed *E. coli* particles after exposure to rFIP-nha WT or N5+39A for 16h in a concentration range from 0.01 µM to 10 µM (n=3). Control cells were exposed to just medium.


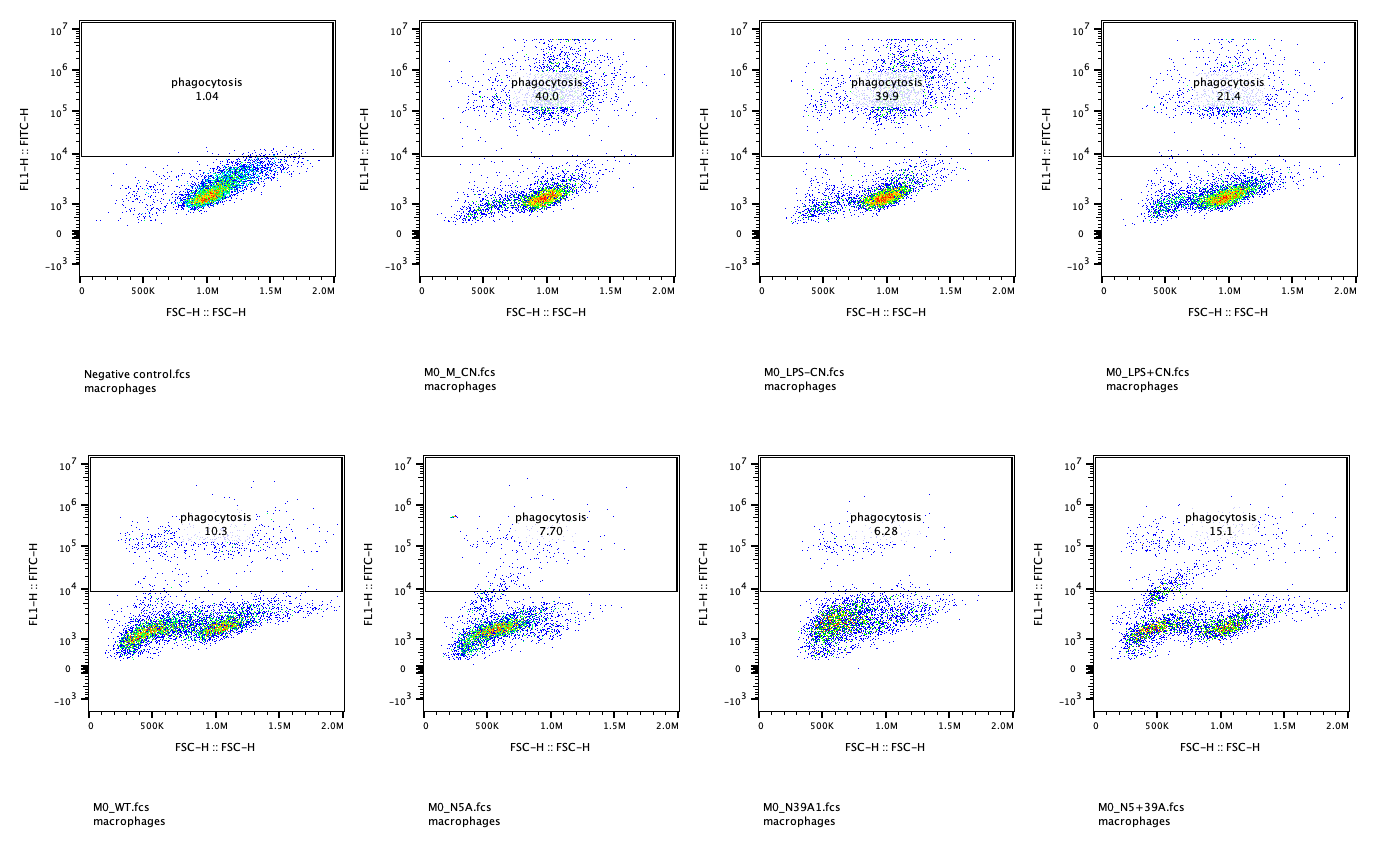


**Figure S6** rFIP-nha decreases THP-1 macrophage phagocytosis of fluorescently labelled *E. coli*. The CytoFlex flow cytometer plots display the percentage of THP-1 macrophages that have phagocytosed E. coli particles after exposure to 10 µM WT, N5A, N39A or N5+39A for 16h (n=1). Control cells were exposed to just medium.


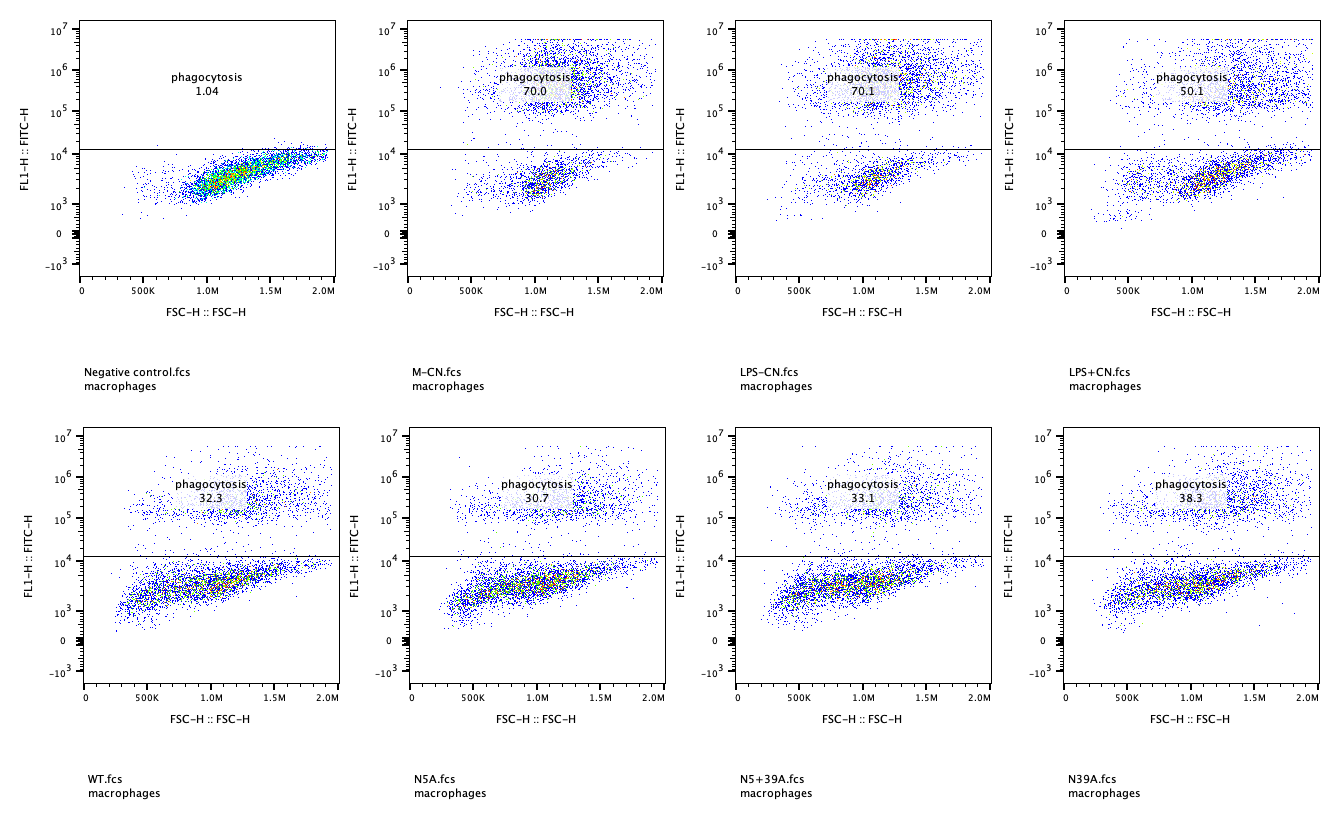


**Figure S7** rFIP-nha decreases THP-1 macrophage phagocytosis of fluorescently labelled *E. coli*. The CytoFlex flow cytometer plots display the percentage of THP-1 macrophages that have phagocytosed E. coli particles after exposure to 10 µM WT, N5A, N39A or N5+39A for 16h (n=2). Control cells were exposed to just medium.


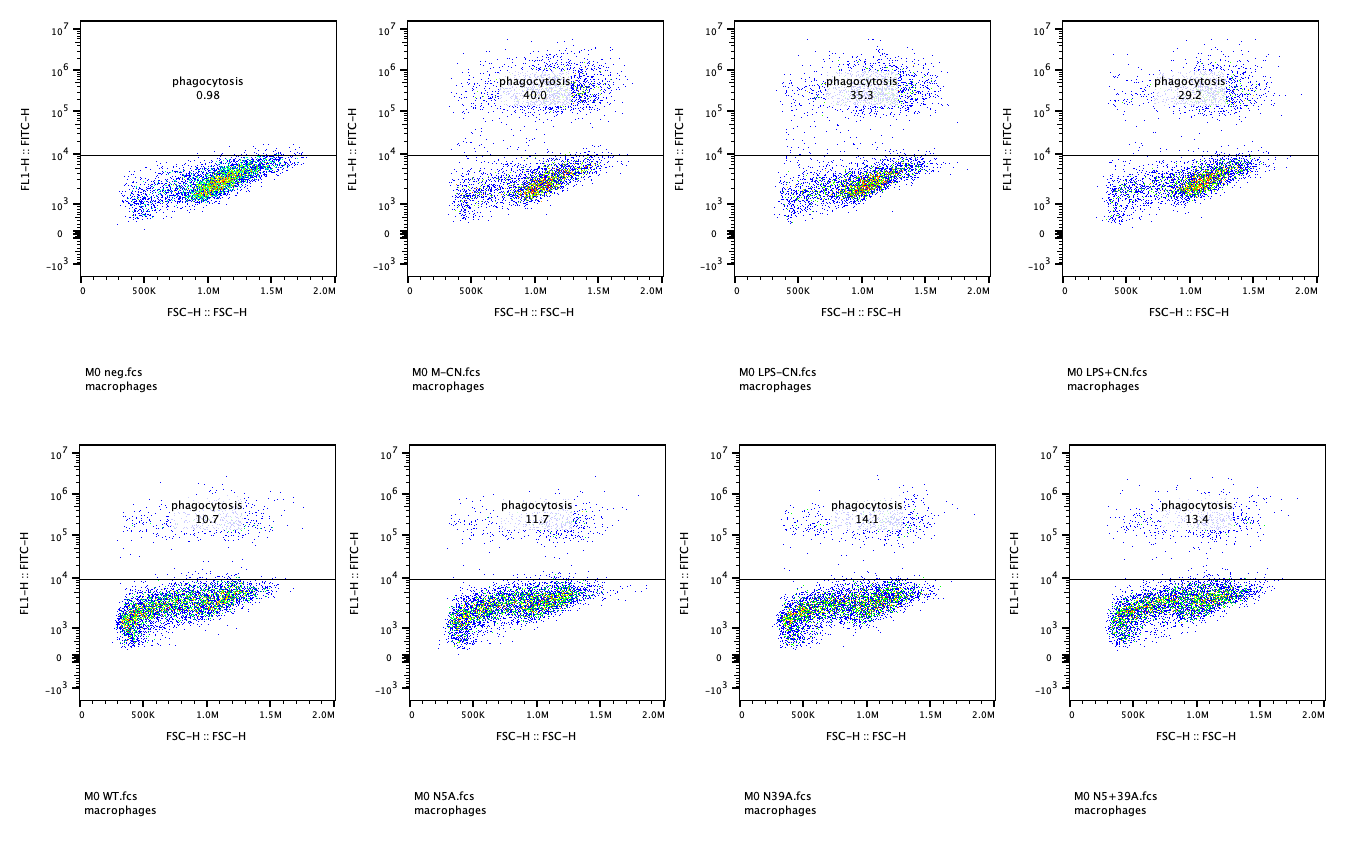


**Figure S8** rFIP-nha decreases THP-1 macrophage phagocytosis of fluorescently labelled *E. coli*. The CytoFlex flow cytometer plots display the percentage of THP-1 macrophages that have phagocytosed E. coli particles after exposure to 10 µM WT, N5A, N39A or N5+39A for 16h (n=3). Control cells were exposed to just medium.

**
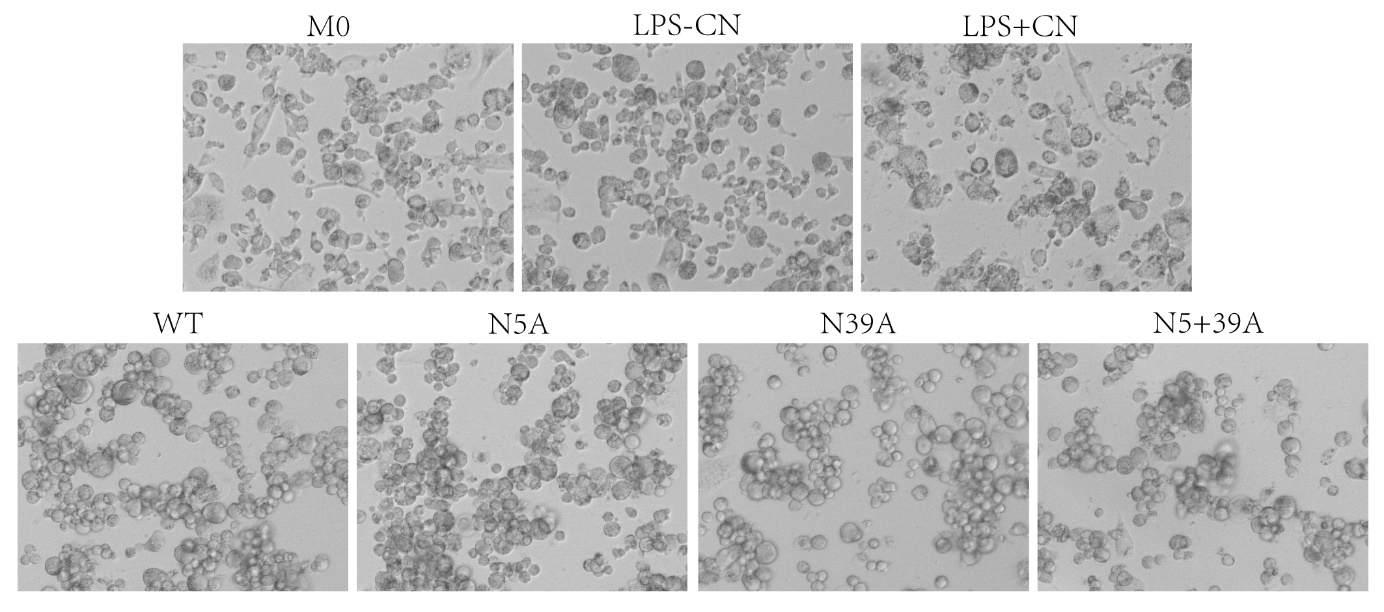
**

**Figure S9** THP-1 macrophage treated with with 10 µM WT, N5A, N39A or N5+39A for 16h. The highest LPS content of rFIP-nha and its mutants was considered as LPS-CN; 1μg/mL LPS was used as LPS+CN.


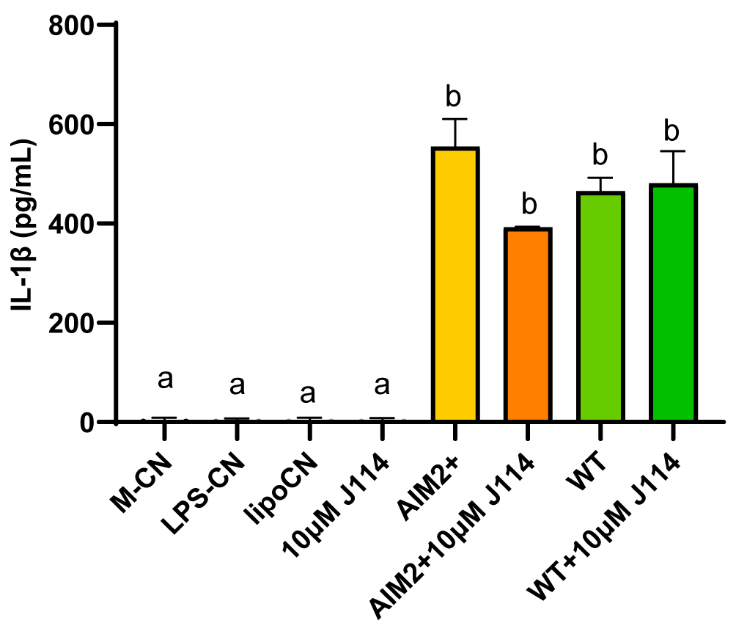


**Figure S10** IL-1β cytokine secretion of THP-1 macrophages treated with AIM2 positive control, rFIP-nha and inhibitor J114 (n=3). The LPS content of rFIP-nha was added as LPS-CN; Lipofectamine^TM^ 2000 was added as lipoCN; 2 μg/mL poly(dA:dT) was transferred to THP-1 macrophages via Lipofectamine^TM^ 2000 as AIM2 positive control (AIM2+). Medium-exposed cells were included as non-treated control (M-CN). THP-1 macrophages were treated with samples for 16h.

# References

1. Carta, S., et al., *The rate of interleukin-1beta secretion in different myeloid cells varies with the extent of redox response to Toll-like receptor triggering.* J Biol Chem, 2011. **286**(31): p. 27069-80.

2. Liu, Y., et al., *Glycosylation Contributes to Thermostability and Proteolytic Resistance of rFIP-nha (Nectria haematococca).* Molecules, 2023. **28**(17).
